# Toward a standard for the evaluation of PET‐Auto‐Segmentation methods following the recommendations of AAPM task group No. 211: Requirements and implementation

**DOI:** 10.1002/mp.12312

**Published:** 2017-07-02

**Authors:** Beatrice Berthon, Emiliano Spezi, Paulina Galavis, Tony Shepherd, Aditya Apte, Mathieu Hatt, Hadi Fayad, Elisabetta De Bernardi, Chiara D. Soffientini, C. Ross Schmidtlein, Issam El Naqa, Robert Jeraj, Wei Lu, Shiva Das, Habib Zaidi, Osama R. Mawlawi, Dimitris Visvikis, John A. Lee, Assen S. Kirov

**Affiliations:** ^1^ Institut Langevin ESPCI Paris PSL Research University CNRS UMR 7587 INSERM U979 Paris 75012 France; ^2^ School of Engineering Cardiff University Cardiff CF24 3AA United Kingdom; ^3^ Department of Radiation Oncology Langone Medical Center New York University New York NY 10016 USA; ^4^ Turku PET Centre Turku University Hospital Turku 20521 Finland; ^5^ Department of Medical Physics Memorial Sloan Kettering Cancer Center New York NY 10065 USA; ^6^ INSERM UMR 1101 LaTIM IBSAM UBO UBL Brest 29609 France; ^7^ Medicine and Surgery Department University of Milano‐Bicocca Monza 20900 Italy; ^8^ Department of Electronics Information and Bioengineering Politecnico di Milano Milano 20133 Italy; ^9^ Department of Radiation Oncology University of Michigan Ann Arbor MI 48103 USA; ^10^ School of Medicine and Public Health University of Wisconsin Madison WI 53705 USA; ^11^ Department of Radiation Oncology University of North Carolina Chapel Hill NC 27599 USA; ^12^ Division of Nuclear Medicine & Molecular Imaging Geneva University Hospital Geneva CH‐1211 Switzerland; ^13^ Department of Imaging Physics MD Anderson Cancer Center Houston TX 77030 USA; ^14^ IREC/MIRO Université catholique de Louvain (IREC/MIRO) & FNRS Brussels 1200 Belgium

**Keywords:** conformity index, outlining assessment, PET/CT, PET segmentation

## Abstract

**Purpose:**

The aim of this paper is to define the requirements and describe the design and implementation of a standard benchmark tool for evaluation and validation of PET‐auto‐segmentation (PET‐AS) algorithms. This work follows the recommendations of Task Group 211 (TG211) appointed by the American Association of Physicists in Medicine (AAPM).

**Methods:**

The recommendations published in the AAPM TG211 report were used to derive a set of required features and to guide the design and structure of a benchmarking software tool. These items included the selection of appropriate representative data and reference contours obtained from established approaches and the description of available metrics. The benchmark was designed in a way that it could be extendable by inclusion of bespoke segmentation methods, while maintaining its main purpose of being a standard testing platform for newly developed PET‐AS methods. An example of implementation of the proposed framework, named PETASset, was built. In this work, a selection of PET‐AS methods representing common approaches to PET image segmentation was evaluated within PETASset for the purpose of testing and demonstrating the capabilities of the software as a benchmark platform.

**Results:**

A selection of clinical, physical, and simulated phantom data, including “best estimates” reference contours from macroscopic specimens, simulation template, and CT scans was built into the PETASset application database. Specific metrics such as Dice Similarity Coefficient (DSC), Positive Predictive Value (PPV), and Sensitivity (S), were included to allow the user to compare the results of any given PET‐AS algorithm to the reference contours. In addition, a tool to generate structured reports on the evaluation of the performance of PET‐AS algorithms against the reference contours was built. The variation of the metric agreement values with the reference contours across the PET‐AS methods evaluated for demonstration were between 0.51 and 0.83, 0.44 and 0.86, and 0.61 and 1.00 for DSC, PPV, and the S metric, respectively. Examples of agreement limits were provided to show how the software could be used to evaluate a new algorithm against the existing state‐of‐the art.

**Conclusions:**

PETASset provides a platform that allows standardizing the evaluation and comparison of different PET‐AS methods on a wide range of PET datasets. The developed platform will be available to users willing to evaluate their PET‐AS methods and contribute with more evaluation datasets.

**List of abbreviations**
AAPM: American Association of Physicists in Medicine
AL: Agreement Limits
AT: Adaptive thresholding
BRENPHN: Brest Numerical Phantom H&N data
BRENPLU: Brest Numerical Phantom Lung data
CERR: Computational Environment for Radiotherapy Research
CT: Computed Tomography
DICOM: Digital Imaging for COmmunications in Medicine
DICOM‐RT: DICOM extension to RadioTherapy
RTSTRUCT: DICOM‐RT structure data set
DSC: Dice Similarity Coefficient
DUV: Delineation Uncertainty Volume
FBP: Filtered Back‐Projection
FLAB: Fuzzy Locally Adaptive Bayesian statistical segmentation method
FT40: Fixed Thresholding at 40% maximum intensity
FT42: Fixed Thresholding at 40% maximum intensity
FT50: Fixed Thresholding at 50% maximum intensity
GATE: Geant4 Application for Tomographic Emission
GATE SIM: GATE Simulation
GCM: Gaussian Clustering Model
GMM: Gaussian Mixture Model clustering
GTV: Gross Tumor Volume
GUI: Graphical User Interface
HD: Hausdorff Distance
H&N: Head and Neck
KM: K‐means clustering
MILPPAB: Milan Physical Phantom Abdominal data
MRI: Magnetic Resonance Imaging
OSEM: Ordered Subset Expectation Maximization
PET: Positron Emission Tomography
PET‐AS: PET‐Automatic Segmentation
PETASset: PET‐AS Suite of Evaluation Tools
PETSTEP: PET Simulator of Tracers via Emission Projection
PPV: Positive Predictive Value
PSF: Point Spread Function
RC: Reference Contour
RG: Region‐growing
DP: Discriminative Power
SBR: Signal to Background Ratio thresholding
SD: Standard Deviation
SUV: Standardized Uptake Value
TG211: Task Group 211 of the AAPM
UCLPTLU: UCL patient Lung data
UCLPTHN: UCL patient H&N data
VOI: Volume Of Interest
WC: Watershed‐based Clustering
WT: Watershed

## 1 Introduction

Positron emission tomography (PET) shows great potential for improving outcomes in cancer patients.^1^ This functional imaging modality provides information that can be used for a variety of clinical applications including patient staging and prognosis, radiation therapy planning, therapy monitoring, and the detection/prediction of recurrences or metastatic disease.^2^, ^3^, ^4^, ^5^ For all these purposes, accurate delineation of the functional tumor volume in PET is of great importance, and the need for reliable PET‐auto‐segmentation (PET‐AS) methods has been widely expressed. However, despite the abundance of developed approaches, there is currently no established agreement on the most reliable technique for routine clinical PET‐AS use. In addition, there are currently no universally established protocols or benchmarks for comparative performance evaluation of such PET‐AS methods for clinical use.

In this context, the report of the American Association of Physicists in Medicine (AAPM) Task Group 211 (TG211)^6^ found that the selection of a single method among those available is a challenging task considering the large number of published PET‐AS algorithms and the variability of methodological approaches and their associated level of validation. The task group acknowledged the need for developing a standard evaluation framework (benchmark) designed for the assessment of both existing and future PET‐AS algorithms. The report also pointed out that the value of a benchmark would rely heavily on the choice of testing data, as well as on the associated performance evaluation metrics.

In this work, we describe the requirements for the design and implementation of such benchmark and report on the PET‐AS Suite of Evaluation Tools (PETASset) package which was developed in line with the recommendations of TG211.

## 2 Materials and methods

In this section, we propose recommendations for standard features of the benchmark. These can be grouped according to (a) usability and accessibility, (b) application areas, and (c) performance criteria.

### 2.A. Usability and accessibility

In order for a standard to be usable, it is essential that the software is easy to use and quick to learn, although it is safe to assume some level of prior knowledge in the field (e.g., PET image analysis and segmentation) from the users. In particular, the user interface is required to be intuitive and accompanied by comprehensive documentation to guide the user through common useful cases or specific tasks. In addition, the software is required to be accessible to the public and understandable by the targeted user‐base. It may be desirable also that the design allows the software to be further extended and used for other applications in the future.

### 2.B. Application areas

The areas of application of the benchmark relate to the field of oncology. Image types are expected to reflect the state‐of‐the‐art in diagnostic imaging and treatment management and to adopt the most recent digitized histopathology methods and bespoke phantoms. The benchmark should be easily extendable to satisfy the needs of more application areas according to the availability of new data and new technology. The types of Volumes of Interest (VOI) included in a standardized evaluation protocol should at a minimum include disease sites established for using PET in radiotherapy treatment planning.

Best estimates of reference contours (RC) may originate from various sources depending on the image type included in the dataset. We distinguish between the following types of RC:

- Absolute truth: only available for simulated images.
- Single ‘best’ estimates: surrogate of truth provided for physical phantom images and in the special case of patient images for which histopathology data are available. In physical phantom images, the optimal threshold in simultaneous CT images provides a uniquely best estimate but cannot be considered the absolute truth because of threshold uncertainty arising from partial volume effects and potential misalignments between PET and CT datasets. The accuracy of RC data for patient scans provided by pathology examination of excised lesions is limited due to specimen deformation during processing and possible metabolic changes between the time of scan and the time of excision.^7^
- Multiple equally ‘best’ estimates: they can be provided in the form of consensus manual expert delineations when no single delineation can be considered to be the best.

### 2.C. Performance criteria

This section describes the outputs expected from a benchmark in terms of both segmentation results and subsequent analysis using quantitative metrics extracted from the images. The benchmark is required to evaluate the agreement of PET‐AS results with the best available ‘truth’ estimate, as well as their robustness and the clinical implications of segmentation inaccuracies. The term ‘agreement’ relates to both volumetric and geometric properties. This is in line with the end‐points defined in the TG211 report, which includes “the spatial distribution of the tracer obtained from the PET image after correcting for physical artifacts” (cf. Ref. 6, section 4.A). Performance criteria for segmentation methods can include:^6^, ^8^

- Accuracy: ability to recover the true tumor contour
- Reproducibility: ability to provide the same result when used multiple times on the same image
- Efficiency: ability to minimize computational complexity and workflow
- Robustness: ability to provide similar results under varying acquisition and image reconstruction conditions

In the case of PET‐AS methods that rely on a pure automatic approach without user intervention, the reproducibility is expected to be 100%, and the efficiency including human and computational resources required for the segmentation is expected to be high due to the automatic process. Hence, it is suggested that the benchmark evaluation tools should focus on accuracy and robustness of the PET‐AS methods. Following these requirements, the accuracy metrics to be included in the benchmark are grouped into three categories, corresponding to increasing degrees of complexity:

- Level I: metrics that assess the agreement in terms of volumetric properties such as the number of voxels in the VOI and the statistics of PET signal integrated over that volume
- Level II: metrics that quantify the geometric agreement including spatial matching between a particular PET‐AS contour and the RC
- Level III: metrics that evaluate the clinical relevance of the disagreement between PET‐AS contours and RCs. These metrics describe the “knock‐on” effect that segmentation inaccuracy has on parameters used in treatment selection and planning and, in the case of external‐beam radiotherapy, dose delivery. This functionality is expected to ultimately be related to treatment outcome and is not implemented in the first release of this benchmark.

The robustness metrics should include, as suggested by Hatt et al.*,* 9 the analysis of the sensitivity to the following variations:

- across datasets, governed by differences in anatomy and physiology as reflected by the image characteristics,
- within a dataset, resulting from natural differences in gross tumor volume shape/size between different patients, and
- within an image, according to differences in image reconstruction and noise levels across different realizations of that image.

### 2.D. Recommendation for standard requirements

The following components and functionalities are desirable for the benchmark software:

**A1**: Open access to the software and functionalities that are understandable by both *developers* and *users* of the segmentation methods.
**A2**: Collection of datasets representing the clinical applications requiring validation of PET-AS methods.
**A3**: Carefully selected images and RCs checked to only include cases that can provide meaningful metric values.
**A4**: Capability to allow future extensions by adding new datasets.
**A5**: Implementation of PET-AS methods representing the current state-of-the-art, as described in ref. 6.
**A6**: Capability to facilitate the addition of an algorithm that is developed outside the application's framework and that can be used to segment images and produce contours in a format compatible with the benchmark analysis routines.
**A7**: Metrics to quantify the agreement between PET-AS volumes and RCs and to evaluate the clinical implications of segmentation inaccuracies.
**A8**: Functionalities allowing the evaluation of robustness of PET-AS methods.
**A9**: Ability to directly compare the accuracy of the segmentation of:
  - the same image using different PET‐AS methods
  - different images using the same PET‐AS method.

### 2.E. Evaluation of the benchmark

The evaluation of the benchmark aims at addressing the following question: how good are the chosen datasets and metrics at quantifying and comparing the performance of PET‐AS methods? The implicit hypothesis is that the collection of images and metrics provided with the benchmark is appropriate and allows comparing PET‐AS methods with enough accuracy and precision to distinguish between the methods and identify their specific strengths and weaknesses.

To validate this hypothesis, we need to test both the Discriminative power (DP) and specificity of the benchmark for differentiating PET‐AS methods. DP, which here relates to the ability to distinguish between two PET‐AS methods that are close in performance, can be tested by evaluating the range of variation of the performance metrics across the range of PET‐AS methods and images. Specificity, defined here as the ability of the software to detect changes in performance that are linked to the difficulty of the segmentation, can be tested by comparing reference contours with a modified version of these contours which were prepared to be less accurate.

## 3 Results

This section summarizes the approach taken to implement the standard with the PETASset software while satisfying aims **A1** – **A9** and the recommendations given in Section Methods. The PETASset code was written in the Matlab language (The Mathworks Inc, Natick, USA), including the Image Processing Toolbox. PETASset was implemented as a plugin to the Computational Environment for Radiotherapy Research (CERR) software.^10^ This enabled using CERR's capabilities for handling and displaying Digital Imaging and Communications in Medicine (DICOM) data, as well as dealing with Radiotherapy Treatment (DICOM‐RT) data.

PETASset reference data are stored and distributed in a Matlab structure saved in the CERR file format, and are compatible with all the tools available in CERR. This format is also readable by any other Matlab‐based application. An application programming interface that can be used to read Matlab formatted data in external environments is also available and is described elsewhere.^1^ PETASset is freely distributed along with CERR, for which user documentation is easily accessible,^2^ in line with **A1**.

**Figure 1:**
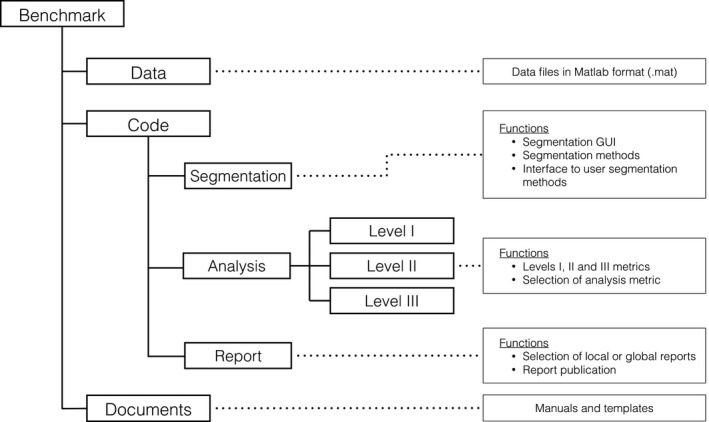
PETASset package structure and content.

The folder structure of the PETASset package and a short description of its content are given in Fig. 1. A detailed description of PETASset's content and functionalities is given in the following section.

### 3.A. Data

#### 3.A.1. Datasets

With reference to Fig. 1, the “Data” folder contains the image datasets (cases) distributed with PETASset. These are provided in compressed CERR file format. The cases include phantom and patient scans for three anatomical sites: H&N, lung, and pelvis. Reference contours were generated using (a) histopathology specimen, (b) simulation templates, or (c) another imaging modality (e.g., CT). The reference contours are considered to be the best estimate of RCs for the cases provided with PETASset. A total of 66 PET studies with RCs from four different research centers are currently included in the PETASset database.

The cases included in each dataset are reported in Table 1 and described in more details below:

- UCLPTLU^11^: 10 clinical PET/CT lung cases, with two PET scans corresponding to different spatial sampling, i.e., different voxel sizes.
- UCLPTHN^12^: seven clinical PET/CT H&N cases.
- MILPPAB^13^: 11 cases corresponding to successive acquisitions of the same physical body phantom.
- BRENPLU^14^: two simulated PET lung study generated with the GATE simulation toolkit.^15^
- BRENPHN^14^: six simulated PET H&N cases generated with the GATE simulation toolkit.
- SIM^16^: a total of 30 simulated PET scans, 10 for each of the sites: pelvis (SIMPTAB), lung (SIMPTLU), and H&N (SIMPTHN). Each dataset contains two reconstructions using the Ordered Subset Expectation Maximization (OSEM) algorithms and OSEM + point spread function (PSF) correction, for five different simulated structures with different geometry and location. These data were generated with the PET Simulator tool described in III.C.4.

**Table 1:** PETASset benchmark datasets

Dataset	Reference	Center	Data type	Anatomical region	Number of studies	Number of series/study	Number of structures/series	Reference contour	CT data	Additional features
UCLPTLU	Wanet et al.^11^	Université catholique de Louvain	Patient	Lung	10	2	1	Specimen	Yes	2 voxel sizes/PET scan
UCLPTHN	Daisne et al.^12^		Patient	H&N	7	1	1	Specimen	No	–
MILPPAB	Zito et al.^13^	Fondazione IRCCS Ca’ Granda Ospedale Maggiore Policlinico	Phantom	Lung & Pelvis	11	6	1	CT	No	Different acquisition instances
BRENPHN	Hatt et al.^14^	LaTIM, INSERM	Phantom	H&N	6	1	1	Simulation	No	Heterogeneous (2 RC contours)
BRENPLU			Phantom	Lung	2	1	1	Simulation	No	Heterogeneous (2 RC contours)
SIMPTLU	Berthon et al.^16^	MSKCC/Cardiff University	Patient	Lung	10	5	1	Simulation	No	5 RC geometries/ 2 reconstructions/ 5 acquisition instances
SIMPTHN			Patient	H&N	10	5	1	Simulation	No	5 RC geometries/ 2 reconstructions/ 5 acquisition instances
SIMPTAB			Patient	Pelvis	10	5	1	Simulation	No	5 RC geometries/ 2 reconstructions/ 5 acquisition instances

The cases were chosen in line with requirement **A2,** with the inclusion of both clinical PET/CT used in state‐of‐the art treatment management and state‐of‐the‐art phantom data. According to requirement **A8**, this set of cases was selected to allow testing the robustness of the different PET‐AS methods included in PETASset to:

- different reconstruction parameters for the same patient/phantom (UCLPTLU, SIM)
- different acquisitions, with different Signal to Noise Ratio, of the same physical phantom (MILPPAB)
- different instances of simulated VOIs, generated according to the selection of different parameters controlling the image reconstruction process (BRENPHN).
- different VOI geometries and locations for the same underlying normal PET uptake (SIM).

The target volumes were chosen in line with requirement **A2**, with a focus on lung and H&N cancer. Cutting‐edge histopathology and tissue heterogeneity data, modeled in the simulated datasets (BRENPLU, BRENPHN, SIM) were also included.

#### 3.A.2. Reference contours

In PETASset RCs are hidden from the user and are only used for evaluation purposes.

With reference to Table 1, the RCs included in the current version of PETASset are

- UCLPTHN: 1 RC per series. The contour was derived from the macroscopic specimen obtained after surgery, digitized on a flatbed scanner and registered to the CT scan.^11^
- UCLPTLU: 1 RC per series. The contour was derived from the macroscopic specimen obtained after surgery, digitized on a flatbed scanner and registered to the CT scan. The same RC is used for both reconstruction types.^12^
- MILPPAB: 1 RC per series, for 11 different zeolite tumor models positioned in various regions of the physical phantom and six different acquisition instances. Reference contours were derived from thresholding on the corresponding CT, iteratively adapted to fit the known volume.^13^
- BRENPLU and BRENPHN: 1 RC per series, encompassing the whole tumor even in case of heterogeneous uptake. These contours correspond to the tumors defined in the original simulation map.^14^
- SIM data: 1 RC contour per series, extracted from the original simulation PET uptake map.^16^

**Figure 2:**
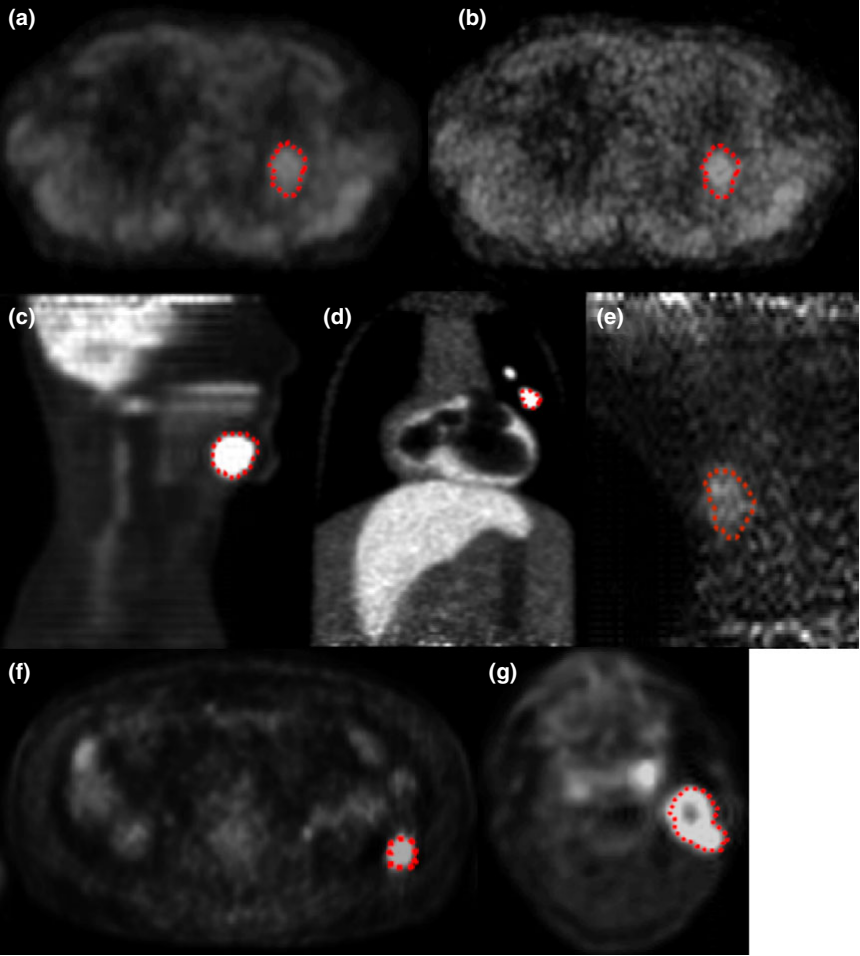
Examples of PET images and RCs available in PETASset. (a) and (b) UCLPTLU, same lesion with different image resolution; (c) BRENPHN; (d) MILPPAB; (e) UCLPTHN; (f) SIMPTAB; (g) SIMPTHN (cf. Table1). [Color figure can be viewed at wileyonlinelibrary.com]

Figure 2 shows examples of the PET images available in PETASset including the associated RCs.

### 3.B. Workflow and analysis

**Figure 3:**
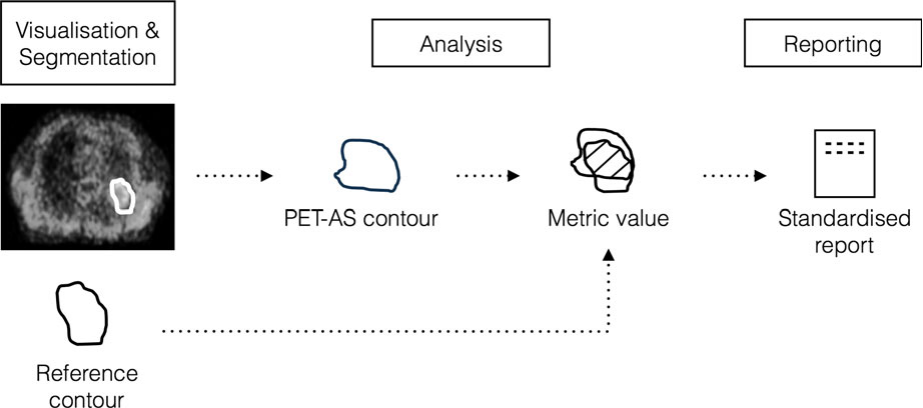
PETASset workflow for a given study, RC and PET‐AS method. [Color figure can be viewed at wileyonlinelibrary.com]

The workflow implemented in PETASset is shown in Fig. 3 and includes:

- Image and VOI visualization
- Image segmentation
- Data analysis and evaluation of performance metrics
- Structured reporting

The input to PETASset is a set of contours the accuracy of which has to be evaluated. The contours can be generated using the (a) default PET‐AS methods provided with PETASset, (b) the segmentation module available in CERR or (c) custom Matlab code (cf. Fig. 1).

**Figure 4:**
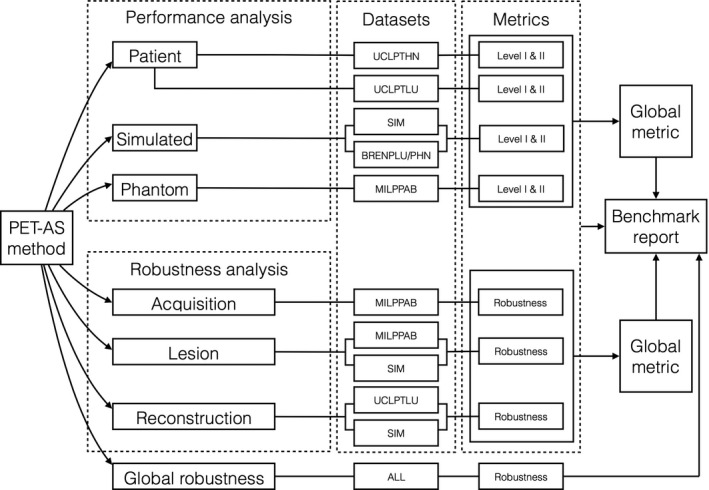
Analysis workflows implemented in PETASset.

A number of different analyses can be carried out in PETASset as shown in Fig. 4. Depending on the used dataset and evaluation metric, a given PET‐AS method can be tested in terms of absolute accuracy and/or in terms of robustness to a specific reconstruction parameter. For instance, testing a PET‐AS method on UCLPTLU data with Level I and II metrics provides a quantitative measure of its performance on clinical lung tumor data. In addition, the robustness of a PET‐AS method to different image acquisitions of the same subject can be assessed using the MILPABB dataset as the standard deviation of results obtained for the same lesions over the different acquisition instances available.

All evaluation strategies, represented by Level I‐II metrics, operate on three types of data:

- PET image
- User‐generated contours
- Reference contour

It is worth noting that in PETASset all RCs and PET‐AS contours in each study are defined in the same frame of reference and in the same co‐ordinate grid as the image space and resolution of the PET data are used. The evaluation strategies implemented in PETASset are described in Section 3.C.2.

### 3.C. Code

With reference to Fig. 1, the PETASset code system is saved in the “Code” folder. All the code files (or functions), written in the Matlab language, are accessible from the PETASset drop‐down menu and are separated into three categories (a) Segmentation, (b) Analysis, and (c) Report described in detail in the next sections.

#### 3.C.1. Segmentation

The functions in the Segmentation category are used by the PETASset segmentation tool to segment images and generate contours. The PETASset segmentation tool is accessible through a Graphical User Interface (GUI) which allows visualization and segmentation of the current PET scan. Once the PET scan is selected, PETASset automatically displays axial, sagittal, and coronal views of the volume on which the RC is defined. The segmentation algorithm can be then chosen from a drop‐down list. The list includes the PET‐AS methods provided by default with the PETASset package plus any additional custom algorithm implemented by the user. The result of the image segmentation can be displayed and saved together with the information on algorithm and parameters used. The PET‐AS segmentation methods implemented by default in PETASset include:

- fixed threshold (percentage of maximum)
- fixed threshold (absolute threshold)
- region growing
- statistical clustering

The methods were selected from common thresholding and advanced methods as defined by Hatt et al.^6^ (sections 2.B.1 and 2.B.2). It is worth noting that custom segmentation methods coded in the Matlab language can also be used as well as any manual or threshold methods implemented in the current version of CERR or indeed any third‐party PET‐AS method that supports file export to the DICOM RTSTRUCT format.

#### 3.C.2. Analysis

The functions in the Analysis category implement metrics used for the evaluation of the agreement between PET‐AS‐generated contours and the RC. The code files are grouped in two folders: Levels I and Level II, which correspond to different metrics that can be launched independently from the PETASset menu. The PETASset analysis tool is also accessible through a GUI that allows the user to select the PET‐AS contours and the metrics to use in the study. The results of the analysis are automatically saved to file and can also be shown in tabular format. Level I and II metrics are described in the next sections.

##### Level I

Level I metrics are used to provide basic and essential information on the delineated VOI. The metrics include:

- Volume
- Mean uptake value
- Maximum uptake value
- Centre of mass

Each Level I metric can be used to quantify the deviation between the PET‐AS and the RC. Uptake statistics and absolute volume are scalar quantities and the deviation from the reference value is given by the signed fractional difference. The center of mass is a vector quantity and the deviation from the reference value is given by the Euclidean distance between PET‐AS and centers of mass of the RC. If we denote with A and B, the set of voxels delimited by the RC and by the PET‐AS contours respectively, we can define the following errors for Level I metrics:

(1) $$∙\;error\;in\;volume=\frac{\left(|B|-|A|\right)}{\left|A\right|}\times 100\;\left(\%\right)$$

(2) $$\begin{array}{c} ∙\;error\;in\;mean\;uptake=\frac{\left(mean\;uptake\;(B)-mean\;uptake\;(A)\right)}{mean\;uptake\;(A)}\times 100\;\left(\%\right) \end{array}$$

(3) $$\begin{array}{c} ∙\;error\;in\;maximum\;uptake=\frac{\left(maximum\;uptake\;(B)-maximum\;uptake\;(A)\right)}{maximum\;uptake\;(A)}\times 100\;\left(\%\right) \end{array}$$

(4) $$\begin{array}{c} ∙\;error\;in\;center\;of\;mass=\|center\;of\;mass\;(B)-center\;of\;mass\;(A)\| \end{array}$$

where || and ‖‖ represent set cardinality and the Euclidean norm, respectively.

##### Level II

Level II metrics are used to quantify the similarity between the PET‐AS and the RC. This similarity can be expressed in terms of geometrical properties and spatial overlap. The following metrics were implemented in PETASset:^6^

- Dice Similarity Coefficient (DSC)

(5) $$DSC\;\left(A,B\right)=\frac{2\times |A\cap B|}{\left|A|+|B\right|},\;\text{range}\;\left[0,1\right]$$

- Sensitivity (S)

(6) $$S=\frac{\left|A\cap B\right|}{\left|A\right|},\;\text{range}\;\left[0,1\right]$$

- Positive Predictive Value (PPV)

(7) $$PPV=\frac{\left|A\cap B\right|}{\left|B\right|},\;\text{range}\;\left[0,1\right]$$

- Modified Hausdorff Distance (HD)^17^

(8) $$\begin{array}{cc} & HD=\text{max}\left(\frac{1}{N_{A}}\sum_{i}min_{j}\|a_{i}-b_{j}\|,\frac{1}{N_{B}}\sum_{i}min_{j}\left\|b_{i}-a_{j}\right\|\right),\\ & \;\text{range}\;[0,+\infty ) \end{array}$$

- Delineation Uncertainty Volume (DUV)

(9) DUV=(|A∪B|)-(|A∩B|),range[0,+∞)

where A and B are the set of voxels delimited by the RC and by the PET‐AS contours respectively, | | represent set cardinality, and ‖a−b‖ is the Euclidean distance of point *a* on the RC to point *b* on the PET‐AS contour. The range of values that can be achieved is indicated for each metric. The metric in Eq. 8 is a variant of the Hausdorff distance (averages replaces the maximum). This formulation was implemented in PETASset following the results of Dubuisson et al.^17^ showing its superiority compared to the original algorithm in quantifying the similarity between two contours. The DUV in Eq. 9 is the cardinality of the volume between the reference and test surfaces. From DUV, one can derive the average delineation uncertainty in voxels (or cm knowing the voxel dimensions) as the average thickness of this volume.

An example of Level II analysis performed on a single study (1 RC) of the UCLPTLU dataset is given in Table 2 for metrics DSC, S, PPV, and HD. The PET‐AS methods were: Watershed‐based clustering (WC), Signal to Background Ratio (SBR) thresholding method as described by Geets et al.^18^ and fixed thresholding with 40% and 50% of the maximum tumor intensity (FT40 and FT50 respectively). In this case, the RC was extracted from a digitized macroscopic specimen (cf. UCLPTLU in Table 1). Values obtained for the RC correspond to the best metric value achievable.

**Table 2:** Example of Level II analysis using RC data from a single series in the UCLPTLU dataset and different PET‐AS methods

Method	DSC	S	PPV	HD (cm)
RC	1	1	1	0
WC	0.778	0.754	0.804	0.250
SBR	0.642	0.511	0.864	0.318
FT40	0.652	0.525	0.861	0.318
FT50	0.469	0.315	0.920	0.378

#### 3.C.3. Report

The functions in the Report category are used by the reporting tool to produce structured reports that summarize the results of the PETASset analysis. PETASset supports two types of report: “Local” and “Global”. Both reports can be generated through dedicated GUIs. PETASset report supports different output formats (html, pdf, or doc document), which can also be selected by the user. Both reports are described in more detail in the following paragraphs.

##### Local Report

The Local Report is designed to summarize the performance of PET‐AS methods for a single study and a selection of metrics. The structured report contains the following sections:

- PETASset analysis details:

- Name of the image file corresponding to the selected study
- List of PET‐AS contours selected
- List of metrics used in the analysis

- Level I analysis:

- Table of Level I metric values for the selected PET‐AS contours
- Graphs of the values obtained across PET‐AS contours for each metric

- Level II analysis:

- Table of Level II metric values for the selected PET‐AS contours
- Graphs of the values obtained across PET‐AS contours for each metric

##### Global Report

The Global Report is designed to include the performance of PET‐AS methods across several cases. It allows one or more PET‐AS methods to be evaluated and ranked using different performance metrics across the whole benchmark dataset. The Global Report also provides additional statistics data such as the mean and standard deviation of metric values, for each dataset across all the selected cases. It can also be used for longitudinal studies.

The structure of the Local and Global Reports is the same, except for one additional section which contains the following items:

- A table reporting the mean and standard deviation of each metric value across the selected PET‐AS contours
- A table containing mean metric values across cases within each dataset separately
- Graphs showing the mean and standard deviation of each metric value across cases within the different datasets grouped by data type (clinical, nonclinical) and tumor site.

#### 3.C.4. 3D PET simulator

The 3D PET simulator PETSTEP^3^^,^ 16 was also implemented in PETASset. With PETSTEP synthetic 3D PET scans can be generated using the PET or CT data provided with PETASset. Tumors of any shape, maximum SUV, and tracer uptake distribution can be added to the original PET or CT image. Different scanner and reconstruction parameters can also be set by the user. Currently implemented reconstruction techniques include Filtered Back‐Projection (FBP), and OSEM algorithms with or without Point Spread Function (PSF) modeling.^19^ The PETSTEP functionalities allow users to generate reference PET and RC data that can be used to test and optimize their own segmentation methods and/or to test the robustness of PET‐AS methods to a particular image parameter, reconstruction setting, or acquisition instance.

#### 3.D. Evaluation of the implementation

In line with the evaluation objectives defined in section 2.E, we assessed the DP of PETASset to distinguish eight PET‐AS methods including:

- FT42: fixed threshold of 42% maximum intensity
- FLAB: fuzzy locally adaptive Bayesian statistical segmentation method^20^
- GMM: Gaussian Mixture Model clustering^21^
- AT: Adaptive thresholding^22^
- RG: Region‐growing^22^
- KM: K‐means clustering^22^
- GCM: Gaussian Clustering Model^22^
- WT: Watershed^22^

Contours were obtained outside PETASset for the methods not implemented in the software, such as FLAB and GMM.

Level I and Level II analyses were carried out using the PETASset functionalities described in Section 3.C.2. Table 3 reports the average Level I and Level II metric values calculated across all RCs with associated standard deviation. The median and standard deviation across all methods is also reported at the bottom of the table, together with the range. The standard deviation (SD) of Level I metrics across PET‐AS methods, given with the median value in Table 3, ranged between 40% (absolute error in volume) and 94% (error in maximum SUV value) of the median value, corresponding to values of 17 and 1.6, respectively. For level II metrics, SDs ranged between 8.4% (PPV) and 20% (S) of the median value, corresponding to values of 0.07 and 0.13, respectively.

**Table 3:** Average Level I and Level II metric values calculated across the entire PETASset dataset and associated standard deviation

Method	Level I Absolute metric error (% RC)			Level II			
	Volume	Max SUV	Mean SUV	DSC	S	PPV	HD (cm)
FLAB	27 ± 15	3.0 ± 12	6.3 ± 11	0.74 ± 0.07	0.69 ± 0.09	0.82 ± 0.09	0.25 ± 0.16
GMM	21 ± 25	5.0 ± 11	0.21 ± 10	0.76 ± 0.08	0.77 ± 0.08	0.78 ± 0.09	0.17 ± 0.12
FT50	60 ± 37	0.89 ± 11	3.7 ± 35	0.53 ± 0.08	0.43 ± 0.11	0.91 ± 0.10	0.30 ± 0.08
FT42	61 ± 70	0.36 ± 9.8	15 ± 20	0.64 ± 0.07	0.56 ± 0.09	0.88 ± 0.09	0.24 ± 0.08
RG	42 ± 21	0.18 ± 12	11 ± 18	0.68 ± 0.07	0.62 ± 0.10	0.85 ± 0.11	0.23 ± 0.10
KM	70 ± 163	2.7 ± 11	11 ± 58	0.73 ± 0.10	0.85 ± 0.05	0.69 ± 0.13	0.27 ± 0.20
GCM	39 ± 13	0.98 ± 9.6	9.0 ± 17	0.70 ± 0.06	0.65 ± 0.09	0.83 ± 0.09	0.19 ± 0.05
WT	42 ± 26	2.5 ± 11	3.3 ± 18	0.67 ± 0.07	0.63 ± 0.11	0.79 ± 0.10	0.22 ± 0.08
Range	21/70	0.18/5.00	0.21/15	0.53/0.76	0.43/0.85	0.69/0.91	0.17/0.30
Median (SD)	42 (± 17)	1.7 (± 1.6)	7.7 (± 4.9)	0.69 (± 0.07)	0.64 (± 0.13)	0.83 (± 0.07)	0.24 (± 0.04)
Agreement limits (example)	(0,59)	(0,3.3)	(0,12.6)	(0.62,1)	(0.51,1)	(0.76,1)	(0,0.28)

PETASset can also provide, for each metric, Agreement Limits (ALs) to indicate the range of values that can be expected by a new segmentation method compared to the performance of existing PET‐AS methods already evaluated with PETASset. ALs for example could be defined as the minimum and maximum values of a range corresponding to one standard deviation centered on the median value (Table 3) or by confidence limits as determined from future research. It is worth noting that for metrics such as DSC, S, and PPV that provide a finite measure of agreement, the upper limit of the AL should be set to the maximum achievable value.

**Figure 5:**
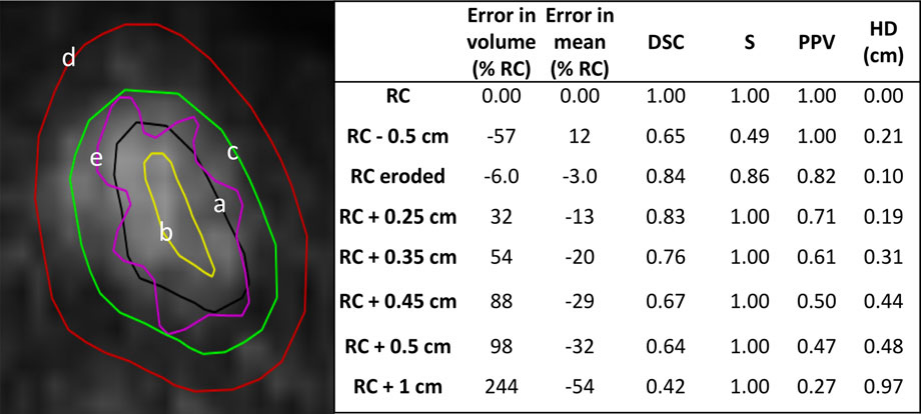
Specificity analysis for the UCLPTHN test case. (a) RC: black, (b) ‘RC 0.5 cm’: yellow, (c) ‘RC + 0.5 cm’: green, (d) ‘RC + 1 cm’: red, and (e) ‘RC eroded’: magenta. Other contours are not shown for the sake of clarity. [Color figure can be viewed at wileyonlinelibrary.com]

The specificity analysis was carried out by modifying the PETASset RCs to introduce known inaccuracies, on one of the UCLPTHN series. The following test contours were generated to represent typical segmentation errors due to under‐contouring, over‐contouring and different contour shape:

- isotropic shrinkage of 0.5 cm (RC – 0.5 cm)
- isotropic expansion of x = 0.25, 0.35, 0.45, 0.5, 1.0 cm (RC + x cm)
- iso‐volumetric erosion/dilation (the RC was eroded and dilated locally to modify its geometry while maintaining the same volume)

where expansions of 0.5 and 1.0 cm were used to model “moderate” and “large” over‐contouring, respectively. All modified contours were compared to the RC in terms of volumetric error, error in mean, DSC, S, PPV, and HD. The results of this analysis are given in Fig. 5.

Results of the specificity analysis on Fig. 5 provide clear rankings of the different contours for the different metrics considered. In terms of the error in volume for example, the smallest error is obtained for RC eroded, which was designed to have a volume very close to the RC. The largest error is obtained by RC + 1 cm, which is also visually the contour the most different from RC. The sign of the error in volume also provides information on the type of segmentation error (over‐segmentation or under‐segmentation). Because the metrics chosen provide complementary information, the respective rankings are different.

The variations observed across methods and test cases are considered large enough to conclude that PETASset provides informative results for the comparison of PET‐AS methods, thereby validating the discriminative power of PETASset. In addition, PETASset is able to accurately and clearly rank contours with known and different inaccuracies, for example small variations in volume, which validates its specificity. It should be noted, however, that the specificity will be limited by the accuracy of the volume contour definition in CERR. Furthermore, the specificity, as described here, will vary with the RC size: larger RCs are expected to lead to smaller differences between metric values when testing volumes of similar known volume error.

The results given in Table 3 are an illustration of how ALs could be defined, based on the hypothesis that datasets and PET‐AS methods implemented in PETASset are representative of the current state‐of‐the art. In this example, with reference to Table 3, a PET‐AS method would be within the ALs for the volumetric error if its absolute mean error in volume across the PETASset VOIs was lower than 59% of the true volume. It would be within the ALs for the DSC, if its mean DSC across the PETASset was between 0.62 and 1. However, the ALs provided in this work should not be used in practice as they were provided only as an illustration of the PETASset's capabilities. Further investigation is needed to systematically assess clinically relevant and acceptable ALs for the metrics considered in this study.

## 4 Discussion

### 4.A. Design and implementation

PETASset was designed and built following AAPM TG211 report which identified the need for developing a standard evaluation framework designed for the assessment of both existing and future PET‐AS algorithms including those derived from supervised machine learning methods.^6^, ^23^, ^24^ Figure 4 shows the workflows available in PETASset and the large range of information that can be extracted from the current version of the benchmark. It is important to note that, although a number of datasets and evaluation metrics are included, PETASset is *not* required, and therefore not designed, to evaluate each PET‐AS method on all datasets using all metrics. Such a requirement is unrealistic due to different assumptions made about the datasets by different PET‐AS methods and metrics. For example, a PET‐AS method may operate on hybrid data, which assumes the availability of both PET and CT datasets. This assumption may hold for a patient dataset but not for numerical or physical phantoms. The imaging and RC data available in the current version of PETASset are intended to represent the state‐of‐the‐art and have been assembled with the contribution of leading clinical and research institutions in the field. The PETASset datasets include RCs for homogeneous (physical and numerical phantoms) as well as heterogeneous tracer uptake (numerical phantom and clinical data). However, we emphasize that in order to be meaningful, the comparison of PET‐AS methods developed with the same goal should be performed on the same datasets and according to the same metrics.

We therefore recommend that the overall performance of a PET‐AS method is evaluated on all the PET‐ASset data applicable, for a given segmentation method. This can be done via the automated analysis tool embedded in the software, which can select the analysis to be carried out according to the segmentation type. For more specific tests, such as robustness to a particular image reconstruction method, users can follow the process shown in Fig. 4.

The design of PETASset allows users to evaluate segmentation methods themselves. This can be done either by importing segmentation contours produced externally, or by adding a segmentation tool to the benchmark software.

Level II analysis data are the primary output of PETASset since they are easy to interpret and compare between PET‐AS methods and since spatial accuracy is a main concern. Level I metrics provide essential information which put Level II results into context and allow users to make additional considerations regarding the relevance of the observed contouring errors.

PETASset can provide ALs for all evaluation metrics included in the package. This can be used to compare the performance of new and well established PET‐AS methods. The quality and usefulness of these ALs will depend on the quality of data available in PETASset, which will need to be regularly updated. It should be noted that the ALs provided in this document are not recommended for the evaluation of new PET‐AS, since they were derived using a small number of PET‐AS methods and a limited set of images. Further work is needed to produce task‐dependent and reliable ALs for PET image segmentation.

The RC is hidden via encryption in PETASset when the software is distributed to the user. Even then, there is a risk that users optimize their segmentation method blindly to increase the accuracy score of certain algorithms. This optimization may lead to the development of tools that may not perform well outside the package. This risk could be limited by restricting in PETASset the recording of results and the generation of reports to the PET‐AS methods that are tested on all datasets and considering all metrics. Increasing number and diversity of reference images and contours will also help reducing this risk.

Constant improvement and maintenance of the PETASset is needed in order to deal with these challenges.

### 4.B. Future work

The current version of PETASset is a research tool that can be reliably used to evaluate the performance of PET‐AS methods against reference RC data. The following additional functionalities are expected to enhance impact of PETASset in clinical practice:

- Web access: provide web access to PETASset data, tools and statistics including reports for selected datasets and segmentation methods.
- Level III Analysis: design and implement metrics to evaluate the clinical implications of contour accuracy in radiotherapy treatment planning.^25^ It is envisaged that Level III metrics will operate on reference dose maps calculated using PETASset's RC and distributed with the benchmark.
- Reference data: the value of PETASset will be enhanced by adding more test data including 4D PET/CT scans and expert consensus VOIs.^26^ In particular, including images with highly varying degrees of tumor size, activity, contrast and resolution will enable thorough robustness studies in fulfillment of requirement **A8**. It is envisaged that synthetic datasets generated with PETSTEP will also help growing the PETASset database, in particular to include data specifically designed for testing robustness to the partial volume effect.
- Imaging modalities: PETASset should evolve to include the next generation of auto‐contouring methods that combine information from different imaging modalities such as CT and MRI.
- Unified performance score: performance metrics are specific and limited to only certain image or contour parameters. PETASset could be used to combine more metrics in a unified score reporting a single performance value. This would be a desirable feature and research toward such a metric is encouraged.
- Knowledge‐based PET segmentation: continuously adding to the PETASset database standardized data on the performance of different PET‐AS methods will enable us to start building models and ALs to use as a baseline for the assessment of new PET‐AS algorithms and for the optimal segmentation of virtually every type of PET image.

## 5 Conclusions

We presented the methodology followed to develop PETASset, a benchmark dedicated to the standardized evaluation of PET‐AS methods. The benchmark provides a common software platform and state‐of‐the‐art reference data that will be made publicly available. In line with recommendations of AAPM TG211, PETASset addresses the need to provide a framework for an internationally developed standard for the evaluation of PET‐auto‐segmentation approaches.

## Conflicts of interest

The authors have no relevant conflicts of interest to disclose. The paper has not been approved by Science Council and does not represent AAPM guidelines.

## Note

1 Mathworks homepage https://www.mathworks.com/help/matlab/programming-interfaces-for-c-c-fortran-com.html

2 CERR homepage http://www.cerr.info

3 PETSTEP is available for download from: https://github.com/CRossSchmidtlein/PETSTEP
